# CR3 Engaged by PGL-I Triggers Syk-Calcineurin-NFATc to Rewire the Innate Immune Response in Leprosy

**DOI:** 10.3389/fimmu.2019.02913

**Published:** 2019-12-17

**Authors:** Émilie Doz-Deblauwe, Florence Carreras, Ainhoa Arbues, Aude Remot, Mathieu Epardaud, Wladimir Malaga, Véronique Mayau, Jacques Prandi, Catherine Astarie-Dequeker, Christophe Guilhot, Caroline Demangel, Nathalie Winter

**Affiliations:** ^1^ISP, Infectiologie et Santé Publique, INRA, Université de Tours, Nouzilly, France; ^2^Institut de Pharmacologie et de Biologie Structurale (IPBS), Université de Toulouse, CNRS, UPS, BP 64182, Toulouse, France; ^3^Immunobiologie de l'Infection, Institut Pasteur, INSERM U1221, Paris, France

**Keywords:** NFAT, Syk, CR3, phenol glycolipid-1, *Mycobacterium leprae*, dendritic cell, macrophage, neutrophil

## Abstract

*Mycobacterium leprae*, the causative agent of leprosy, is unique amongst human pathogens in its capacity to produce the virulence factor phenolic glycolipid (PGL)-I. In addition to mediating bacterial tropism for neurons, PGL-I interacts with Complement Receptor (CR)3 on macrophages (MPs) to promote infection. We demonstrate here that PGL-I binding to CR3 also enhances bacterial invasion of both polymorphonuclear neutrophils (PMNs) and dendritic cells (DCs). Moreover, in all cell types CR3 engagement by PGL-I activates the Syk tyrosine kinase, inducing calcineurin-dependent nuclear translocation of the transcription factor NFATc. This selectively augments the production of IL-2 by DCs, IL-10 by PMNs and IL-1β by MPs. In intranasally-infected mice PGL-I binding to CR3 heightens mycobacterial phagocytosis by lung PMNs and MPs, and stimulates NFATc-controlled production of Syk-dependent cytokines. Our study thus identifies the CR3-Syk-NFATc axis as a novel signaling pathway activated by PGL-I in innate immune cells, rewiring host cytokine responses to *M. leprae*.

## Introduction

Leprosy, caused by *Mycobacterium leprae* (*M. leprae*) is a chronic infectious disease affecting primarily vulnerable populations in developing countries, with a global prevalence of approximately 200.000 in 2016 (1). While consistently provoking skin lesions with sensory loss, leprosy progression varies extensively across individuals. Patients may develop polar, paucibacillary (tuberculoid), or multibacillary (lepromatous) forms (LL) of the disease correlating with distinctive symptoms and immune profiles (2). Multidrug therapy is highly effective at eliminating bacteria (2). However, treatment often triggers acute inflammatory reactions, such as Type 1 reversal reactions (T1R), or Type 2 Erythema Nodosum Leprosum (ENL) (2, 3) causing severe nerve disabilities. In spite of the different clinical presentations, T1R and ENL share biomarkers such as pro-inflammatory cytokines TNF, IL-1β, or MCP-1 and proteins belonging to the pentraxin family such as C-Reactive Protein in T1R, or pentraxin-3 during ENL (4) suggesting that common immune mechanisms underlie leprosy reactions. Multiple knowledge gaps in the pathophysiology of leprosy still hamper eradication of this complex disease. *M. leprae* is non-cultivable *in vitro*, which is a major hurdle for study of this disease-causing bacterium. Transmission of *M. leprae* remains incompletely resolved and may occur by the respiratory route rather than direct skin-to-skin contact (5). Susceptibility to leprosy is controlled by host genetics and several immune-related candidate genes have been proposed (6). Although Schwann cells are the preferred host niche for *M. leprae*, macrophages (MPs), polymorphonuclear neutrophils (PMNs), and dendritic cells (DCs) have emerged as key players in shaping both protective immunity and immunopathology in leprosy (7). PMNs are a histological hallmark of ENL even though it remains unclear whether they initiate ENL, or are recruited to skin lesions in response to inflammation (3). In LL, MPs are functionally programmed to phagocytose *M. leprae* whereupon they transform into foam cells harboring persistent bacilli, whereas MPs display antimicrobial functions in paucibacillary patients (8). DCs accumulating in LL lesions secrete IL-10 that down-modulates T cells (9). Altogether, these findings support the view that appropriate innate immune responses are critical in the control of *M. leprae* infection.

Pathogenic mycobacteria have evolved sophisticated strategies to establish chronic infections in humans such as the production of a diverse array of lipids and glycolipids with virulence and immunomodulatory properties (10), and *M. leprae* is no exception. Phenolic glycolipids (PGLs) are only produced by mycobacterial species able to persist in the host (11). *M. leprae* produces PGL-I (12) whereas PGL-b is produced by *M. bovis*, including its derivative the Bacillus Calmette-Guérin (BCG) vaccine strain. These molecules share a common lipid backbone and an aromatic nucleus, and are distinguished by their sugar moiety that confers species-specificity. PGL-I plays prominent roles in *M. leprae* virulence: it protects bacilli against the host bactericidal molecules (13), it allows *M. leprae* to colonize peripheral nerves (14), thus damaging them (15), and manipulates the host immune response to the bacterial benefit (16). Using an original approach of genetic reprogramming to produce rBCG::PGL-I as a cultivable surrogate of *M. leprae*, we previously reported that PGL-I targets the lectin domain of Complement Receptor (CR) 3—or integrin αMβ2—to improve bacterial entry into human MPs (17). PGL-I expression also down-regulates the production of NF-κB-dependent cytokines by human MPs, through direct interaction of its trisaccharide domain with Toll-like receptor (TLR) 2 (18). We also observed that PGL-I impaired TRIF-dependent TLR4 signaling, decreasing induction of iNOS in activated MPs (19). While physiologically relevant MPs only represent one arm of the first line of defense against invading *M. leprae*. Our finding that PGL-I promotes CR3-dependent uptake by MPs suggests that other CR3-expressing cells, such as DCs and PMNs (20), are susceptible to *M. leprae* infection. In the present study, we conducted parallel investigations in primary MPs, DCs, and PMNs to gain an integrated view of PGL-I's impact on the innate immune response. Taking advantage of the three cultivable genetically reprogrammed BCG strains (17) that only differ by the ectopic expression—or deletion—of the PGL molecule, we highlight a powerful mechanism of immune deviation evolved by *M. leprae*, as CR3 targeting by PGL-I confers the pathogen with capacity to invade and modulate the cytokine responses of the three cell types. Furthermore, it revealed a novel signaling axis connecting CR3-mediated phagocytosis with Syk-calcineurin-NFAT signaling, bringing a new dimension to immunoregulation in infectious diseases, while providing novel targets for therapeutic intervention in leprosy.

## Materials and Methods

### Bacterial Strains and Growth Conditions

Construction of the recombinant strains rBCG::noPGL, rBCG::PGL-b, rBCG::PGL-I have been described previously (17, 21). The various recombinant strains were transformed with plasmid pWM124, a mycobacterial replicative plasmid carrying the *gfp* gene under the control of the pblaF^*^ promotor (17). Str*ains* were cultured in Middlebrook 7H9 broth (Invitrogen, Cergy-Pontoise, France) containing 0.05% Tween 80 (Sigma-Aldrich, St. Louis, USA) and ADC (5% BSA fraction V, 2% dextrose, 0.003% beef catalase and 0.85% NaCl; BD Microbiology Systems) and supplemented with 40 μg/ml of Kanamycin sulfate (Sigma-Aldrich, St. Louis, USA) or 50 μg/ml of Hygromycin B (Sigma-Aldrich, St. Louis, USA) for the fluorescent strains. Ten days before infection, bacteria were inoculated into 7H9 with ADC without Tween 80. Bacteria were pelleted at 3,000× g 10 min, washed and suspended in PBS. Clumps were dispersed by vortex with 4 mm diameter glass beads. Bacteria were centrifuged (200× g) for 5 min and concentration of bacterial suspensions was measured by OD 600 nm (1 OD = 10^8^ bacilli/ml). To assess CFUs, serial dilutions were plated on Middlebrook 7H11 agar plates supplemented with OADC (ADC supplemented with 0.05% oleic acid).

### Mouse Lines, Ethics Statement and Treatments

Six- to eight-week-old C57BL/6 male mice were obtained from SAS Janvier (Le Genest Saint Isle-France); *itgam*^−/−^ mice were kindly donated by Alain Bessis, and *myd88*^−/−^ mice were bred at Plateforme Infectiologie Experimentale (PFIE, U1277, INRA, Center Val de Loire). Before experiments, all mice were reared at the PFIE in the specific pathogen-free resident animal facility. All animal studies were approved by the “Val de Loire” Ethics Committee for Animal Experimentation (CEEA VdL) and was registered by the French National Committee for Animal Experimentation.

### Mouse Infection

Mice anesthetized by i.p. injection of ketamine/xylazine cocktail received 5 × 10^6^ CFUs of rBCG::PGL-I or rBCG::noPGL under 20 μL in each nostril. Mice received 40 μL of vehicle (DMSO 2%), or 1 μM Syk inhibitor (GS-9973, ApexBio Technology), or 50 ng/ml NFATc inhibitor (Cyclosporin A, Cell signaling Technology) via the nasal route 1 h before and 1 h after BCG infection. Mice were euthanized 24 h post-infection by CO_2_ inhalation.

### PGL-I Binding to Immobilized Receptors and Competition Experiments

Experiments were performed as described (18). Briefly, recombinant mouse or human CR3 (1 μg/well, R&D Systems) were coated on 96-well MaxiSorp™ELISA plates (Nunc) overnight at 4°C. Purified, native PGL-I (BEI Resources, NIAID, NIH) was dissolved in ethanol After washing and blocking, specified concentrations of PGL-I diluted in binding buffer were added to the wells and incubated at 37°C for 1 h. Bound PGL-I was detected by an indirect method using an anti-PGL-I antibody (Ab SC-48, BEI Resources, NIAID, NIH), followed by addition of a secondary horseradish peroxidase (HRP)-coupled goat anti-mouse Ab (BioRad). HRP activity, corresponding to bound PGL-I, was determined by reading the absorbance at 450 nm. For competition assays, purified native PGL-I diluted in ethanol (500 ng/well) was added to 96-well PolySorp™ ELISA plates (Nunc). After evaporation, washing and blocking, recombinant mouse CR3 (500 ng/well) was incubated with 50 μM of synthetic oligosaccharide domains of PGL-b and PGL-I (18), for 1 h at 37°C. CR3 binding to PGL-I coated to the plates was performed as above and bound CR3 was detected by an indirect method using anti-CD11b Ab (2LPM19c, Santa Cruz Biotechnology).

### Bone Marrow and Lung Cells Preparation

Femurs and tibias were harvested from 6-week-old (WT, *itgam*^−/−^ or *myd88*^−/−^) mice reared at PFIE animal facility. Bones from phagocyte-specific *clec7a*^−/−^ mice (line LysM-Cre/*Dectin-1*^L2/L2^ (22) were also kindly donated by Agnes Coste and those from MRP8-Cre^+^*Syk*^*flox*/*flox*^ and LysM-Cre^+^*Syk*^*flox*/*flox*^ by Attila Mocsai (23). Bone marrow-derived cells were obtained as previously described (24). Briefly, DCs were obtained with 1% supernatant from the J558 cell line producing murine granulocyte-macrophage colony-stimulating factor, and MPs were obtained after culture with 30% L929 cell-conditioned medium as a source of macrophage colony-stimulating factor. Two passages were performed in presence of 100 U penicillin and 100 μg/ml streptomycin (Gibco). Cells, used at day 10 for infectivity and cytokine assays, were suspended in complete medium without antibiotics. PMNs were directly purified from bone marrow by magnetic positive selection. Cells suspended in PBS/0.5% FCS were incubated 15 min with purified anti-Ly-6G PE-conjugated antibody (clone1A8, BD Biosciences) followed by 15 min with anti-PE magnetic beads (Miltenyi Biotec). More than 95% pure PMNs were obtained as assessed by microscopy after May-Grünwald-Giemsa staining. Viability by trypan blue exclusion was 98%. Lung cells were collected as previously described (25). Briefly, euthanized mice (*n* = 11–12) were perfused with PBS and lung tissues were digested for 1 h with collagenase D (5 mg/ml, Roche) and DNAse A (40 U/ml, Roche) before filtering cells through 100 μM nylon cell strainer (BD Falcon). For BAL cells and fluid collection, four washes of the lungs with 0.5 ml of cold PBS were performed through cannulated trachea. The first wash was used to measure cytokine while the three other washes were pooled to prepare single cell suspensions that were kept at 4°C until FACS staining. In order to get enough material, BAL was performed on 12 animals of each group and two were pooled (*n* = 5–6). For FACS staining, cells were incubated 20 min with 2% total mouse serum, and then labeled in PBS supplemented with 5% FCS and 0.1% total mouse serum with antibodies against the surface markers CD11b (clone M1/70), Ly6G (clone 1A8), Ly 6C (clone AL-21), all from BD Biosciences.

### Infectivity, Cytokine Assays and Gene Expression Quantification

DCs, PMNs and MPs were infected with rBCG::PGL-I, rBCG::PGL-b, or rBCG::noPGL at MOI of 5 for 2 h at 37°C. As indicated, bacteria were opsonized with 2% of fresh mouse serum and/or 1 μM Syk inhibitor GS-9973 (ApexBio Technology), 1 or 50 nM of Cytochalasin D (Sigma Aldrich), were added 1 h before infection. After three washes in PBS, cells were lysed with PBS containing 0.05% Triton X-100 for 15 min. Dilutions were plated on Middlebrook 7H11 agar supplemented with OADC and CFUs were counted 2–3 weeks later. For cytokine assays, cells were infected as indicated above. After 2 h contact and washes, cells were incubated overnight in complete medium (RMPI, 10% FCS, L-Glutamine). Cells were also stimulated with γ-irradiated *M. leprae*, strain NHDP (BEI Resources, NIAID, NIH) equivalent to MOI 10. To analyze role of the different signaling pathways, cells were treated 1 h before infection with inhibitors of Syk (1 μM of GS 99-73); NFATc (500 pg/ml of FK506-tacrolimus or 50 ng/ml of cyclosporin A; Cell signaling technology); phagocytosis (1 nM or 50 nM of Cytochalasin D). Control wells received DMSO vehicle alone. When indicated, CR3 was blocked by incubating cells for 1 h with 150 μg of anti-CD11b antibody (clone M1/70, BD Biosciences), or Rat IgG2b,κ isotype. Cell culture supernatants were harvested 16–20 h later and cytokines were measured by ELISA with kits (R&D Systems) according to manufacturer's instructions.

WT or *itgam*^−/−^ MPs were infected for 2 h at MOI 5 with the three rBCG strains and total RNA were extracted with the NucleoSpin RNA II kit (Macherey-Nagel). After removal of residual genomic DNA with RNase-free DNase (Macherey-Nagel), RNA quantity and integrity were measured with NanoDrop spectrophotometer (NanoDrop Technologies). Total RNA (1 μg) was reverse transcribed to cDNA using random hexamers and iScript reverse transcription supermix (Bio-Rad) according to the manufacturer's instructions. qRT-PCR was run with iQ SYBRGreen Supermix (Bio-Rad) in a LightCycler^®^ 480 System apparatus (Roche). Reaction mix consisted of 1:10 diluted cDNA in 5 μl nuclease-free water, 300 nM each forward and reverse primer for *il-1*β (for TCTAATGCCTTCCCCAGGGC; rev GACCTGTCTTGGCCGAGGAC) and the three house keeping genes *hprt1* (for CAGTCCCAGCGTCGTGATTA; rev TGGCCTCCCATCTCCTTCAT) *rpl4* (for GACCAGTGCTGAGTCTTGGG; rev GTATTCACTCTGCGGTGCCA) and *ppia* (for GCTGGACCAAACACAAACGG; rev CCAAAGACCACATGCTTGCC) and iQ SYBRGreeen Supermix (Bio-Rad) in a total reaction volume of 15 μl. After 45 cycles of amplification 45 cycles (95°C 5 s; 62°C 5 s) quantification was performed with Bio-Rad Laboratories CFX Manager software. ΔC_q_ values between *il1b* and mean of the three reference genes were calculated as ΔC_q_ = C_q[il1b]_-mean C_q[hprt1, ppia, rpl4]_ to normalize gene expression and ΔΔC_q_ values were calculated between each sample and control as ΔΔC_q_ = ΔC_q[infected cells]_- ΔC_q[mock−infected]_ cells. Data were then expressed as RQ = 2^−ΔΔCq^.

### NFATc Translocation Analysis

MPs derived from bone marrow of WT or *itgam*^−/−^ mice were seeded on 24-well plate coverslips. Adherent cells were infected with rBCG::PGL-I or rBCG::noPGL at MOI 5 for 30 min at 37°C. Cells were fixed in 4% PFA for 20 min. After saturation in D-PBS- BSA with 5% for 30 min, cells were labeled with anti-NFATc2 antibody (clone 25A10.D6.D2, Invitrogen) in PBS containing 0.1% Triton X-100, for 1 h at room temperature. After washings in PBS with 0.001% Triton X-100, cells were incubated with Alexa 633-conjugated goat anti-mouse IgG1 antibody (Invitrogen) for 1 h at room temperature. Slides were mounted with Fluoromount-G medium containing DAPI (Invitrogen). Images were captured with a confocal Leica TCS SP8 microscope. NFATc translocation was quantified by calculating the Manders coefficient using the JaCOP plugin (26) for Image J.

### Statistical Analysis

Data were expressed as arithmetic mean ± standard error of the mean (SEM). Statistical analyses were performed with Prism 4.0 software (GraphPad) and R software (3.4.1 version, Rcmd pluging). Analyzes were performed on data from 2 to 5 independent experiments. Paired non-parametric two-tailed K-Sample Fisher-Pitman Permutation test was used to analyse data, with a Monte Carlo resampling approximation, except for Figure 4E where Mann-Whitney *t*-test was applied. Represented *p*-values are: ^*^p < 0.05; ^**^p < 0.01, and ^***^p < 0.001.

## Results

### CR3 Targeting by PGL-I Allows Broad and Efficient Invasion of Innate Immune Cells

**Figure 1:**
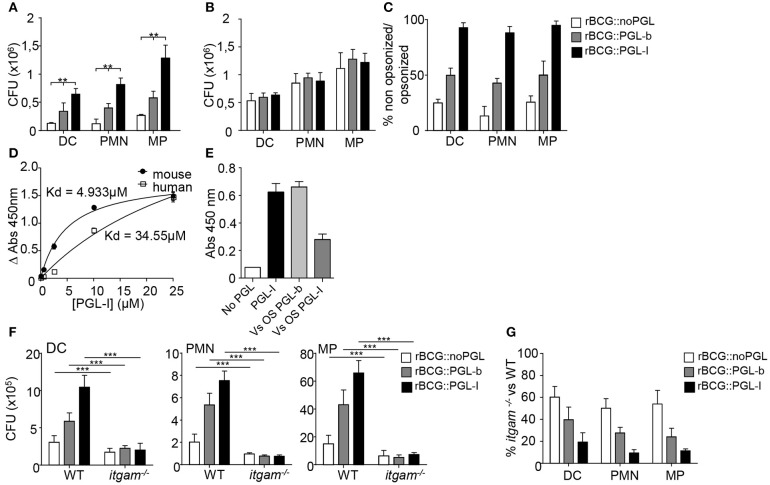
Targeting of the lectin domain of CR3 by rBCG::PGL-I allows efficient infection of innate cells. **(A,B)** Number of CFUs recovered from bone marrow-derived DCs, PMNs and MPs after 2 h infection with rBCG::noPGL, rBCG::PGL-b, or rBCG::PGL-I at MOI of 5 under serum-free conditions (**A**, *n* = 8), or after 30 min complement opsonizing with fresh mouse serum before contact with the cells (**B**, *n* = 4). **(C)** Ratio of CFUs recovered for the three strains under non-opsonizing versus opsonizing conditions. **(D)** Binding of purified native PGL-I to recombinant human or mouse CR3. Absorbance values obtained after indirect ELISA with anti- PGL-I antibodies and determination of the dissociation constant Kd (*n* = 6). **(E)** Competition assay measuring binding of recombinant mouse CR3 to purified PGL-I in presence of the chemically synthesized oligosaccharide domains of PGL-b or PGL-I. Absorbance reveals bound CR3 measured by indirect ELISA with anti-CR3 antibodies (*n* = 3). **(F)** Number of CFUs determined as in **(A)** for the thee strains after infection of DCs, PMNs or MPs derived from wild-type (WT) mice or *itgam*^−/−^ mice deficient in CR3 (*n* = 10). **(G)** Ratio of CFUs recovered from *itgam*^−/−^ vs. WT cells in the three cell types for the three strains (*n* = 10). Data are presented as mean ± SEM. ^**^P < 0.01; ^***^P < 0.001.

We used three genetically engineered BCG strains, reprogrammed to either keep the native PGL-b molecule from *M. bovis* (rBCG::PGL-b), replace it by PGL-I from *M. leprae* (rBCG::PGL-I), or lack expression of any PGL (rBCG::noPGL) (17). Modified BCGs grew comparably and, with the exception of PGL, exhibited a similar envelope (17, 18), making them physiologically relevant tools to dissect the specific role of *M. leprae* PGL-I in mycobacterial interaction with the host. We first compared the infectivity of rBCG::noPGL, rBCG::PGL-b, and rBCG::PGL-I in mouse bone marrow-derived DCs, PMNs and MPs under non-opsonic conditions mimicking primary infection. While rBCG::PGL-b and rBCG::PGL-I both infected the three cell types more effectively than rBCG::noPGL, PGL-I clearly conferred BCG with the highest infectivity (Figure 1A). Since we were interested in the role of CR3 (17), we next analyzed the impact of opsonizing the strains on their phagocytosis. Treatment with fresh serum from naïve mice increased infectivity of all strains to similar levels (Figure 1B). Ratios of bacteria recovered from DCs, PMNs and MPs after infection under non-opsonic conditions vs. serum-opsonizing conditions were around 50% and 20% for rBCG::PGL-b and rBCG::noPGL, respectively (Figure 1C), illustrating the gain conferred by complement opsonization for these two strains. Notably, in all cell types, non-opsonized rBCG::PGL-I infectivity was close to 90% as compared to opsonizing conditions (Figure 1C), emphasizing the role of PGL-I in promoting phagocytosis in all environments and conditions. We previously showed that purified PGL-I efficiently binds human CR3 via its lectin domain (18). Here, we confirmed that PGL-I also bound the mouse counterpart (Figure 1D). Using chemically synthesized oligosaccharide domains of the molecules (18), we observed that PGL-I binding to CR3 was competed out by PGL-I and not PGL-b sugar moiety (Figure 1E) indicating the specificity of the lectin domain of CR3 for the *M. leprae-*specific saccharidic moiety of PGL-I. To evaluate the importance of CR3-mediated phagocytosis in each cell type, we then used bone marrow cells derived from *itgam*^−/−^ mice, which are defective for expression of the CD11b chain in the CR3 heterodimer (27). As compared to their WT counterparts, strain infectivity was decreased in CR3-deficient cells whatever the cell type. This was observed for all strains albeit to different degrees (Figure 1F). Remarkably, infectivity of rBCG::PGL-I was the most affected, with only approximately 10% of bacteria recovered from CR3-deficient cells, as compared to WT (Figure 1G). On the contrary, even though rBCG::noPGL infectivity was less than rBCG::PGL-I in WT cells, half of the bacilli were still recovered from CR3-deficient cells as compared to WT. Therefore, as also found with human MPs (17), production of PGL-I allows mycobacteria to target the CR3 lectin site through its oligosaccharide moiety of the molecule for optimal invasion of DCs and PMNs.

### CR3-Mediated Phagocytosis of PGL-I Expressing Mycobacteria Requires Syk

**Figure 2:**
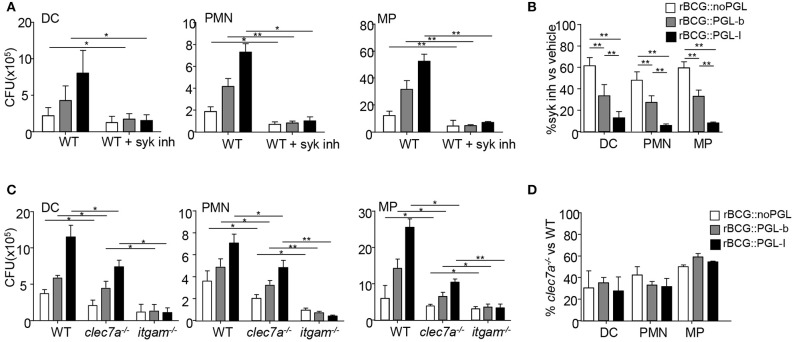
The Syk pathway regulated by CR3 engagement controls infection by rBCG::PGL-I **(A)** Number of CFUs recovered as in Figure 1A from DCs, PMNs, or MPs that were treated with vehicle or 1 μM of Syk inhibitor GS-9973, 1 h before infection with the rBCG strains (*n* = 6). **(B)** Ratio of CFUs recovered from cells treated with the Syk inhibitor vs. vehicle for the three strains and cell types. **(C)** Number of CFUs recovered as in Figure 1A from DCs, PMNs or MPs derived from bone marrow of WT or *itgam*^−/−^ or *clec7a*^−^^/−^ mice harboring the *dectin-1* deficiency specifically in myeloid cells (22) (*n* = 4). **(D)** Ratio of CFUs from *clec7a*^−^^/−^ vs. WT cells for the three strains and cell types. Data are presented as mean ± SEM ^*^P < 0.05; ^**^P < 0.01.

We observed that rBCG::PGL-I infectivity was equally important in opsonized and non-opsonized conditions. The Syk pathway being critical for initiating CR3 integrin signaling (28) and for phagocytosis of opsonized particles (29), we next asked if Syk was involved in effective internalization of rBCG::PGL-I under non-opsonic conditions. Bone marrow-derived DCs, PMNs and MPs were incubated with rBCG::noPGL, rBCG::PGL-b and rBCG::PGL-I in the presence of GS-9973, a selective inhibitor of Syk (30). Syk inhibition reduced the infectivity of all strains into each cell type (Figure 2A). However, this decrease was significantly more important for rBCG::PGL-I, with only 10% of bacteria recovered under Syk inhibition as compared to ~30–50% with rBCG::PGL-b and rBCG::noPGL, respectively (Figure 2B). Interestingly, Syk inhibition and CR3 deficiency induced comparable reduction of rBCG::PGL-I phagocytosis (Figures 1F, 2B). Together, our data thus suggested that Syk critically contributes to the non-opsonic, CR3-mediated phagocytosis of rBCG::PGL-I. Under non-opsonizing conditions, Syk-engagement is well-documented for phagocytosis involving C-type Lectin Receptors (CLRs) other than CR3. For instance Dectin-1 (CLEC7A) (31), which is expressed by DCs, PMNs, and MPs (32), mediates phagocytosis of fungal pathogens via the Syk pathway. Mycobacteria also activate Dectin-1, even though they do not produce β-glucans (33). To evaluate the potential contribution of Dectin-1, we compared infectivity of the three rBCG strains in DCs, MPs, and PMNs from mice bearing selective disruption of the *clec7a* gene in myeloid cells (22), *itgam*^−/−^ and WT mice (Figure 2C). Compared to WT, Dectin-1-deficient cells displayed a comparable decrease in infectivity of the three BCG strains irrespective of their PGL production (Figure 2D). Moreover, infectivity loss of rBCG::PGL-I was less important than in CR3-deficient cells. We conclude that contrary to CR3, Dectin-1 mediates BCG phagocytosis independently of PGL production. Notably, CR3-mediated phagocytosis of rBCG::PGL-I required the Syk pathway in all cell types. Even though Syk-dependent Dectin-1—and possibly other CLRs (34)—cooperated with CR3 for efficient rBCG::PGL-I entry, they did not compensate for the absence of CR3.

### CR3-Mediated Infection by rBCG::PGL-I Selectively Induces Syk-Dependent NF-κB-Independent Cytokines

**Figure 3:**
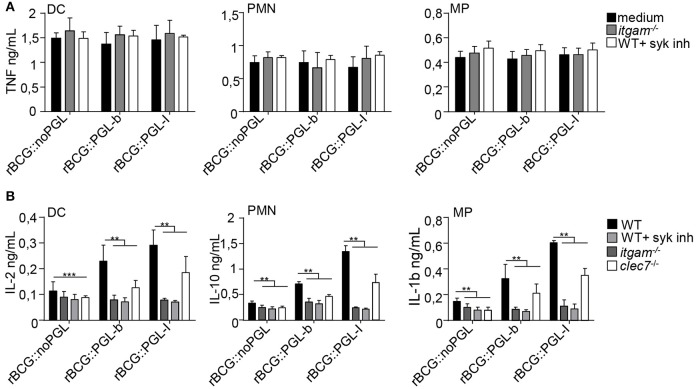
CR3-mediated infection by rBCG::PGL-I induces Syk-dependent NF-κb independent cytokines. **(A)** TNF produced in supernatants of DCs, PMNs or MPs derived from bone marrow of WT mice, treated with DMSO (vehicle), or 1 μM of the Syk inhibitor GS-9973, and *itgam*^−/−^ mice was determined by ELISA after overnight infection with the three rBCG strains at MOI of 5 under non-opsonizing conditions (*n* = 8). **(B)** IL-2 produced by DCs, IL-10 by PMNs and IL-1β by MPs from WT cells treated with DMSO, or 1 μM of the Syk inhibitor GS-9973 or *itgam*^−/−^ or *clec7a*^−/−^ cells infected as in **(A)** were determined by ELISA (*n* = 4). Data are presented as mean ± SEM. ^**^P < 0.01; ^***^P < 0.001.

In response to mycobacteria, Syk participates to pro-inflammatory cytokine gene transcription via activation of the canonical NF-κB pathway in response to various CLR stimuli (33). This pathway, which engages CARD9/Bcl-10/MALT1, comes in addition to classical MYD88-dependent activation of NF-κB that is efficiently triggered by a range of TLR mycobacterial ligands (35). Since Syk was essential for CR3-mediated infection (Figure 1), we tested if PGLs activated Syk-mediated signaling pathways, using NF-κB-dependent and independent cytokines as readouts. As expected in all conditions tested, cells incubated with medium or vehicle alone did not produce appreciable amounts of cytokines (Supplementary Figure S1). After overnight infection, all cell types produced comparable amounts of NF-κB-dependent cytokines, namely TNF (Figure 3A) IL-6 or IL-12p40 (Supplementary Figures S2A,B), whatever the rBCG strain used for stimulation. To our surprise, we did not detect any significant reduction of TNF production by cells derived from *itgam*^−/−^ mice or by WT cells treated with Syk inhibitor (Figure 3A). Therefore, despite being crucial for CR3-mediated infection, Syk signaling did not modify the induction of NF-κB-dependent pro-inflammatory cytokines. Syk activation stimulates the production of other cytokines, such as anti-inflammatory IL-10 in PMNs (36), IL-2 in DCs (37), or IL-1β in MPs (38) (Figure 3B). Notably the production of such cytokines was significantly enhanced by infection with rBCG::PGL-I, compared to the other strains. We measured 2.6-fold more IL-2 produced by DCs, 4.1-fold more IL-10 by PMNs and 4.0-fold more IL-1β by MPs, as compared to rBCG::noPGL. Interestingly, enhanced production of these cytokines was lost in cells derived from *itgam*^−/−^, or in WT cells treated with Syk inhibitor (Figure 3B and Table S1A). In order to rule out potential off-target effects of the Syk inhibitor, we used bone-marrow PMNs from MRP8-Cre+*Syk*^*flox*/*flox*^ mice (23) and MPs from LysM-Cre+*Syk*^*flox*/*flox*^ mice (23) where Syk was cell-specific depleted. Similar dramatic decrease of IL-10 production by PMNs and IL-1β by MPs was observed in response to rBCG::PGL-I in WT cells treated with the Syk inhibitor and in Syk-deficient cells as compared to controls (Supplementary Figure S2C). In MPs, IL-1β is produced under immature form and is then cleaved by the inflammasome (39). We measured if Syk inhibition or CR3 deficiency had any impact on pro-IL-1β gene transcription in MPs. We observed higher levels of pro-IL-1β mRNA synthesis by MPs infected with rBCG::PGL-I as compared to the two other strains. In CR3-deficent MPs or in WT MPs treated with the Syk-inhibitor, pro-IL-1β was reduced (Supplementary Figure S2D) suggesting that IL-1β secretion by MPs by CR3/Syk was regulated at the transcriptional level. The Syk-dependent NF-κB-independent cytokine signature was decreased in Dectin-1 deficient cells as compared to WT, but this was independent of PGL production. On the contrary, this signature was profoundly reduced in cells derived from *itgam*^−/−^ mice in response to rBCG::PGL-I with only 27% of IL-2 produced by DCs, 15% of IL-10 by PMNs and 18% IL-1β by MPs, as compared to WT controls. Of note, Syk inhibition produced the same effects as CR3 deficiency (Figure 3B). By contrast, even though rBCG::noPGL induced lower levels of the three cytokines, CR3 loss or Syk inhibition only reduced their production by half (Table S1A). Therefore, although the Syk pathway could be activated by all strains through Dectin-1, and likely other CLRs (33), maximal production of Syk-dependent NF-κB-independent cytokines observed after infection by rBCG::PGL-I required CR3. For their part, pro-inflammatory cytokines triggered by CARD9/Bcl-10/MALT1 below Syk (33) were not impacted by CR3-mediated infection with rBCG::PGL-I.

### PGL-I-Driven Activation of Syk Triggers Nuclear Factor of Activated T-Cells Nuclear Translocation in Innate Cells and Rewires the Immune Response

**Figure 4:**
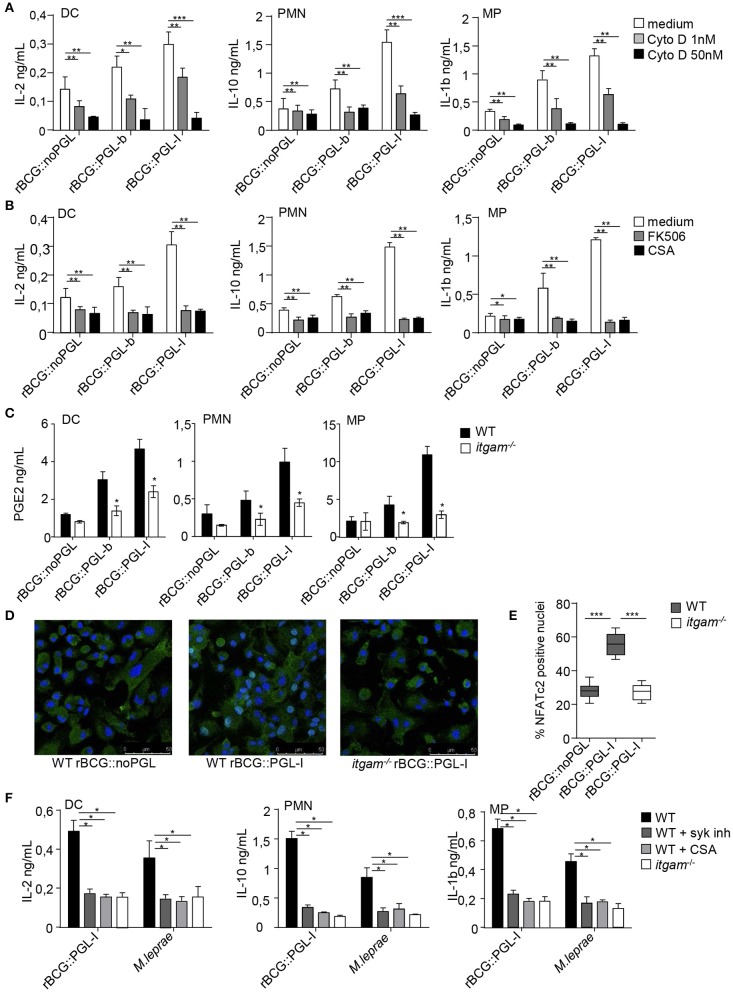
PGL-I targeting of CR3 triggers nuclear translocation of NFATc2 downstream of Syk that controls a specific mediator signature. **(A,B)** IL-2 produced in DCs supernatants, IL-10 in PMNs and IL-1β in MPs were measured by ELISA and **(C)** PGE2 by competition ELISA after overnight incubation the three recombinant BCG strains at MOI of 5. Data are presented as mean ± SEM. ^**^P < 0.01; ^***^P < 0.001. **(A)** Phagocytosis was blocked by treatment for 1 h before infection with 1 or 50 nM of CytoD, while controls received only the vehicle DMSO (*n* = 8). **(B)** Cells were treated with two NFATc inhibitors tacrolimus (FK506, 500 pg/ml) or Cyclosporin (CsA, 50 ng/ml) 1 h before infection by the three rBCG strains. Controls received DMSO (*n* = 4). **(C)** For PGE2 production, cells were derived from bone marrow of WT or *itgam*^−/−^ mice. **(D)** Translocation of NFATc2 (green) into the nucleus of MPs (blue, DAPI staining) derived from bone marrow of WT or *itgam*^−/−^ mice was analyzed by confocal microscopy 30 min after infection with rBCG::PGL-I or rBCG::noPGL at MOI of 5. Cells on slides were then fixed, permeabilized and stained with anti-NFATc2 and mounted in medium containing DAPI. Images were acquired with a confocal Leica TCS SP8 microscope, where NFATc2 colocalization with the nucleus appeared in light blue, while NFATc2 remaining in the cytosol appeared in green. Images are from original magnification ×63. **(E)** After analysis of images (Image J software) the Manders coefficient was determined with the JACoP (26) plugin that allowed to quantify colocalization of NFATc2 with the nucleus (light blue). These coefficients were calculated in 10 fields from 4 slides (obtained from 2 independent experiments). Box and whisker plot shows median ± SEM. ^***^P < 0.001 (Student's *t*-test). **(F)** Cytokine production measured as in **(A,B)** from WT or *itgam*^−/−^ cells stimulated with rBCG::PGL-I at MOI of 5 or γ-irradiated *M. leprae* (strain NHDP, equivalent to MOI of 10). WT cells received treatment with DMSO or Syk inhibitor GS-9973 or CsA to block NFATc translocation, 1 h before contact with bacteria. Data are presented as mean ± SEM. ^**^P < 0.01; ^***^P < 0.001.

Upon recognition of particulate β-glucans by Dectin-1, MPs, and DCs form a phagocytic synapse that activates a signaling cascade involving Syk, calcineurin and Nuclear Factor of Activated T-cells (NFATc) (40). Nuclear translocation of NFATc triggers a specific gene expression program in PMNs and DCs contributing to control fungal infections (41, 42). Having shown that rBCG::PGL-I infectivity was Syk-dependent, we asked if CR3 targeting by PGL-I impacted Syk signaling through the calcineurin-NFATc pathway. We first tested the importance of phagocytosis (40) in this process, by treating cells with the actin polymerization inhibitor cytochalasin D (CytoD) prior to infection with our BCG strains. This treatment had no significant impact on the production of IL-12p40 or TNF (Supplementary Figures S3A,B). On the contrary, CytoD induced a dose-dependent reduction of IL-2 production by DCs, IL-10 by PMNs and IL-1β by MPs (Figure 4A) in response to infection by all rBCG strains. The highest dose of CytoD most dramatically impacted production of cytokines by rBCG::PGL-I infected cells that produced only between 8 and 17% of the amount as compared to vehicle-treated cells, depending on the cell type (Table S1B). This showed that phagocytosis, most efficiently induced by rBCG::PGL-I, was necessary for induction of Syk-dependent NF-κB-independent cytokines. Phagocytosis of particulate β-glucans via Dectin-1 results in calcium mobilization downstream of Syk, which triggers calcineurin-dependent NFATc translocation to the nucleus. NFATc activation is key to instruct IL-2 production by DCs (43). Treatment with NFATc inhibitors cyclosporine A (CsA), or tacrolimus (FK506) reduced the Syk-dependent NF-κB-independent cytokine signature whatever the strain used (Figure 4B and Table S1B). Both treatments most dramatically impacted cytokine production by rBCG::PGL-I infected cells that only retained between 12 and 25% of the amount produced by vehicle-treated controls depending on the cell type (Figure 4B and Table S1B). Another important player in bacterial-induced inflammation is Prostaglandin E2 (PGE2) (44). It is produced following transformation of arachidonic acid by Cyclooxygenase (COX) enzymes. Transcription of inducible COX-2 is under the control of NFATc (45) and as a consequence, the highest production of PGE2 by DCs, PMNs, or MPs was observed in response to rBCG::PGL-I infection. In cells derived from *itgam*^−/^^−^, PGE2 production was reduced, as compared to WT. In absence of CR3, MPs produced similarly low amounts of PGE2 whatever the BCG strain used (Figure 4C). Therefore, in addition to the cytokine signature being under the regulation of NFATc, PGE2 that plays important roles in leprosy (14, 46), was also highly induced by rBCG::PGL-I engaging CR3.

We then analyzed by confocal microscopy NFATc translocation to the nucleus of MPs derived from WT or *itgam*^−/−^ mice (Figure 4D). Shortly after incubation with rBCG::PGL-I we observed localization of NFATc2 to the nucleus in 55% of WT MPs, whereas nuclear translocation was only detected in 25% of *itgam*^−/−^ MPs, a level comparable to that observed in WT MPs infected with rBCG::noPGL (Figure 4E). To see if the immunomodulatory properties of rBCG::PGL-I were conserved for native *M. leprae*, we tested if myeloid cells produced Syk-dependent cytokines in a similar way (Figure 4F). As for rBCG::PGL-I, the three cell types all produced the three signature cytokines after *M. leprae* stimulation. Moreover, CR3 deficiency and Syk or NFATc inhibition reduced IL-2 by DCs; IL-10 by PMNs and IL-1β by MPs to similar extents after stimulation with *M. leprae* or its surrogate rBCG::PGL-I (Table S1C). Thus, CR3 targeting by *M. leprae* PGL-I signals through Syk/calcineurin/NFATc to induce a specific mediator signature.

### Syk/calcineurin/NFATc Is the Preferred Pathway Triggered by rBCG::PGL-I to Rewire Innate Cells

**Figure 5:**
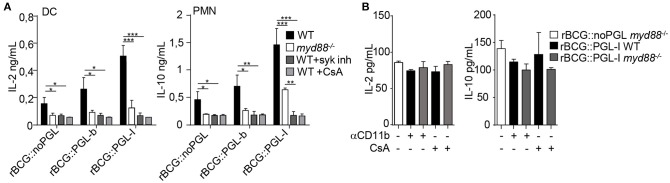
rBCG::PGL-I preferentially triggers Syk/calcineurin/NFATc through CR3 to rewire innate cells **(A)** DCs or PMNs from WT or *myd88*^−/−^ mice were infected with the three rBCG strains at MOI of 5. WT cells were treated with GS-9973 to inhibit the Syk pathway, or CsA to block NFATc translocation 1 h before infection. After overnight incubation supernatants were harvested to measure by ELISA IL-2 produced by DCs and IL-10 produced by PMNs. **(B)** Before infection of DCs or PMNs with rBCG::noPGL or rBCG::PGL-I as in **(A)**, cells were either incubated for 1 h with anti-CD11b antibody M1/70 to block CR3-mediated entry, or exposed to CsA to block NFATc translocation as indicated. IL-2 produced by DCs and IL-10 by PMNs after overnight incubation were measured by ELISA. Data are presented as mean ± SEM (*n* = 4). ^*^P < 0.05; ^**^P < 0.01; ^***^P < 0.001.

NFATc activation triggered by Dectin-1 in response to particulate β-glucans is not connected to TLR activation (41, 43). However, MYD88 seems crucial for IL-10 production by mycobacteria-infected PMNs (36). We queried then if MYD88 was compulsory for production of the Syk/calcineurin/NFATc cytokine signature downstream of CR3. In agreement with Zhang et al. (36), MYD88-deficient PMNs produced less IL-10 than WT, irrespective of the strain used for infection. We also observed that MYD88-deficient DCs produced less IL-2, as compared to WT (Figure 5A). MYD88^−/−^ cells infected with rBCG::noPGL produced minimal levels (Supplementary Figure S3C), even though amounts were above the background measured in cells with medium alone (Supplementary Figures S1D,E). On the contrary, rBCG::PGL-I infected MYD88^−/−^ cells retained 27% of IL-2 (DCs), or 44% of IL-10 (PMNs) production, as compared to their WT counterparts (Figure 5A). As expected, production was reduced to minimal levels (i.e., similar to rBCG::noPGL-infected MYD88^−/−^ cells) when Syk or NFATc translocation was blocked (Figure 5A). This showed that Syk-dependent NFATc translocation upon rBCG::PGL-I targeting of CR3 did not depend on MYD88 to induce the specific cytokine signature. We then blocked CR3 or NFATc translocation in rBCG::PGL-I-infected WT and MYD88-deficient cells. In both case, cytokine production was reduced to the minimum levels observed with rBCG::noPGL infected MYD88-deficient cells (Figure 5B) indicating that the MYD88 pathway was not able to rescue the Syk/NFATc pathway when the mycobacterium producing PGL-I targeted CR3. This clearly showed that CR3/Syk/NFATc was preferentially triggered by rBCG::PGL-I independently of MYD88, even though the two pathways cooperated to induce maximum levels of the signature cytokines.

### CR3 Targeting by PGL-I Signals Through Syk/NFATc *in vivo*

**Figure 6:**
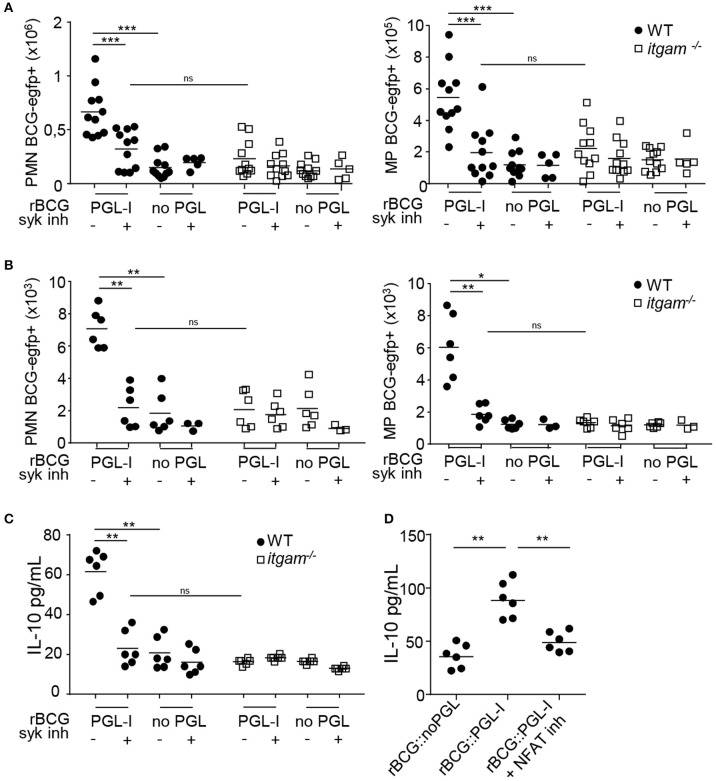
rBCG::PGL-I targeting CR3 triggers Syk and NFATc *in vivo*. **(A,B)** WT and *itgam*^−/−^ mice were nasally infected with 5 × 10^6^ CFUs of fluorescent rBCG::PGL-I or rBCG::noPGL, and received two nasal doses of Syk inhibitor GS-9973 administered 1 h before and after bacteria. BAL and lung tissues were harvested 24 h later to analyze cells by flow cytometry. **(A,B)** Numbers of Ly-6G^+^, CD11c^−^ PMNs and Ly-6G^−^, CD11c^+^ MPs harboring BCG-EGFP^+^ recovered from the lung parenchyma from 11 individuals **(A)** or BAL from 12 individuals pooled per 2 **(B)**. **(C)** IL-10 produced *in situ* by lung cells was analyzed by ELISA in the first BAL from 12 individuals pooled per 2. **(D)** WT mice received two nasal doses of CsA 1 h before and 1 h after rBCG::PGL-I or rBCG::noPGL inhalation, to block NFATc translocation. IL-10 produced *in situ* by lung cells was analyzed as in **(C)**. Data are represented as individual values from *n* = 11 **(A)** or *n* = 6 **(B–D)** from two independent experiments. ^**^P < 0.01; ^***^P < 0.001.

To confirm that PGL-I also targeted CR3 *in vivo*, we infected WT or *itgam*^−/−^ mice with rBCG::PGL-I. Fluorescent versions of rBCG::PGL-I, and rBCG::noPGL as control, were used to track infected cells. Since aerosol infection is suspected for *M. leprae* transmission (5, 47) we chose the intranasal route to administer bacteria, Syk inhibitor, or vehicle controls. Moreover, CD11b -component of CR3- that is overexpressed by inflammatory mouse and human alveolar MPs (48, 49) may represent an important target of PGL-I *in vivo*. Twenty-four hours post-infection, cells from the lung parenchyma or bronchoalveolar lavage (BAL) were harvested and analyzed by flow cytometry for infection of Ly-6G^+^, CD11c^−^ PMNs and Ly-6G^−^, CD11c^+^ cells that were mostly alveolar MPs (50) (see Supplementary Figures S4A–E for gating strategy) in lungs (Figure 6A), or BAL (Figure 6B). Neither Syk inhibition, nor CR3 deficiency impacted the number of PMNs or MPs recruited to the lung (Supplementary Figures S4C,F). However, a higher incidence of infected PMNs and MPs was detected after infection with rBCG::PGL-I as opposed to rBCG::noPGL, showing the enhancing effect of PGL-I on mycobacterial phagocytosis by host cells *in vivo*. On the contrary, in PMNs and MPs harvested from the lungs of *itgam*^−/−^ mice, the frequencies of rBCG::noPGL and rBCG::PGL-I uptake were similar. Furthermore, entry of rBCG::PGL-I into PMNs and MPs in mice receiving the Syk inhibitor was comparable to mice infected with rBCG::noPGL. These data confirmed *in vivo* the key roles of (i) PGL-I in targeting CR3 and (ii) the involvement of the Syk pathway to allow operational CR3-mediated entry into myeloid lung cells. We then measured IL-10 in BAL (Figures 6C,D), where PMNs were the most abundant (Supplementary Figure S4F). In WT mice infected with rBCG::PGL-I, IL-10 production was 2.9-times more elevated than in mice receiving rBCG::noPGL (Figure 6C). In contrast, both strains induced similar levels of IL-10 in BAL from *itgam*^−/−^ mice, or mice treated with the Syk inhibitor. The enhanced production of IL-10 in BAL of rBCG::PGL-I-infected mice thus likely reflects increased phagocytosis by PMNs, leading to CR3-mediated activation of the Syk signaling cascade. To evaluate the role of NFATc in this process, we treated WT mice with two doses of CsA 1 h before and 1 h after inhalation of rBCG::PGL-I or rBCG::noPGL and measured IL-10 produced in BAL 24 h later (Figure 6D). As expected, we observed higher levels of IL-10 in BAL from mice infected with rBCG::PGL-I, as compared to rBCG::noPGL. However, treating rBCG::PGL-I -infected mice with CsA reduced IL-10 produced in BAL to levels comparable to rBCG::noPGL-infected mice, indicating that NFATc translocation is involved in PGL-1-driven enhanced production of IL-10 (**see Figure 6B**). Together, these data confirmed the ability of PGL-I to target CR3 for potent phagocytosis by PMNs and MPs *in vivo*, resulting in activation of the Syk/calcineurin/NFATc pathway and enhanced production of IL-10 by PMNs.

## Discussion

**Figure 7:**
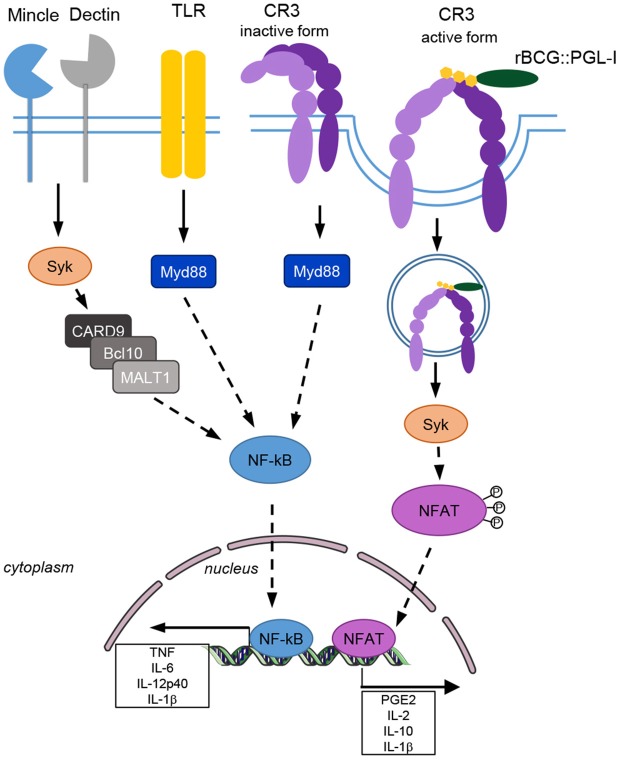
CR3 triggers the Syk/calcineurin/NFATc pathway upon engagement by PGL-I-producing mycobacteria to rewire the innate response. rBCG::PGL-I targets the lectin domain of CR3 on the surface of DCs, PMNs, and MPs. Dectin-1 and CR3 cooperate to induce highly efficient entry of the bacilli. This triggers Syk for translocation of NFATc to the nucleus and initiates a transcriptional program to generate an NF-κB-independent mediator signature. This preferentially PGL-I-triggered pathway does not depend on MYD88 even though both cooperate to induce maximum levels of these mediators.

We discovered that production of the lipid virulence factor PGL-I endows *M. leprae* and recombinant BCG with the unique capacity to engage CR3 for potent phagocytosis in three major subsets of innate cells: DCs, PMNs, and MPs (Figure 7). This efficient phagocytosis resulted in Syk-dependent NFATc translocation to the nucleus that rewired cells to produce a NFATc-specific signature of soluble mediators including IL-2 by DCs, IL-10 by PMNs, IL-1β by MPs and PGE2 by the three cell types. This Syk and NFATc biological signature was also observed in response to native irradiated *M. leprae* and *in vivo* in the lungs of mice after intranasal infection with rBCG::PGL-I. In addition to our previous findings on the key ability of PGL-I-producing mycobacteria to disable TLR2 (18) and TLR4 (19) pathways, our present findings highlight a newly discovered NFATc pathway, efficiently triggered by PGL-I engaging CR3, as an additional weapon that *M. leprae* uses to fine tune the host response. Indeed, the NFATc family of transcription factors has long been recognized as central to T-cell development and functions (51). It is one of the most important targets to control rejection of solid organs allografts. NFATc triggering appeared more recently as a key regulator of both pro and anti-inflammatory processes (52) governed by innate cells. It's considered a hallmark of successful initiation of innate responses to particulate antigens (53). NFATc translocation is controlled by the Ca^2+^/calmodulin phosphatase calcineurin that responds to intracellular increases in Ca^2+^ upon formation of the phagocytic synapse in MPs and DCs. This occurs when particles are engulfed and allows innate cells to distinguish microbes from soluble compounds to trigger an appropriate antimicrobial cell response (40). This important checkpoint was discovered with non-opsonic phagocytosis of β-glucans particulate forms upon binding of the receptor Dectin-1 (40). We observed that Dectin-1 deficiency in DCs, PMNs, and MPs (32) resulted in decreased BCG phagocytosis. However, this was independent of PGL production. For their part, PGLs modulated infectivity upon binding of the complement receptor CR3. Remarkably, rBCG::PGL-I infectivity under non-opsonic conditions was as efficient as after opsonizing with complement. During *Candida albicans* infection Dectin-1 is involved in inside-out signaling that activates CR3 to engage phagocytosis by PMNs (54). Our data show effective collaboration between these two receptors for phagocytosis of rBCG::PGL-I. We propose that Dectin-1—and possibly other CLRs- deliver the first signal for phagocytosis independently of PGL. Then, binding of the sugar moiety of PGL-I to activated CR3 reinforces the phagocytic synapse that efficiently triggers NFATc nuclear translocation.

The signaling pathway leading to nuclear NFATc translocation is controlled by the pleiotropic hub Syk. During mycobacterial infection, the Syk-CARD9 pathway is essential to control bacilli multiplication and overwhelming inflammation (55). Syk controls cytokine genes transcription via activation of the canonical NF-κB pathway below recruitment of the CARD9/BCL-10/MALT1 complex. For mycobacteria Syk is triggered by the C-type lectin Mincle targeted by the cord factor, by Dectin-1 recognizing unknown ligands (33) and potentially by others such as Dectin-2, Mcl, or DCAR. It was recently discovered that IL-2 production by DCs (40, 43), and IL-10 by PMNs (41), involves Syk-dependent elevation of Ca^2+^ through action of PLCγ that leads to NFATc nuclear translocation. While depending on Syk, this cascade does not lead to association with CARD9 and is also different from TLR signaling. Thus, Syk/NFATc induces a different transcriptional program compared to the Syk/NF-κB, or MYD88/NF-κB pro-inflammatory programs (56). So far, activation of NFATc was only reported when fungi, or particulate β-glucans signal through Dectins (40, 43), and LPS through CD14 (57). We identified the lectin-receptor CR3 as another trigger of NFATc translocation to the nucleus when engaged by PGL-I-bearing mycobacteria. Accordingly, we observed dramatically reduced IL-2 production by DCs and IL-10 by PMNs upon blockade of NFATc by tacrolimus or CsA. Zhang et al. previously reported that coactivation of Syk and MYD88 was necessary to allow IL-10 production by PMNs infected with BCG, or the *M. tuberculosis* H37Rv lab strain (36). We observed that Syk-controlled NFATc translocation triggered by PGL-I-producing mycobacteria, could also induce IL-10 production by PMNs and IL-2 by DCs independently of MYD88. We observed that NFATc blockade dramatically reduced IL-1β production by rBCG::PGL-I, or *M. leprae*-infected MPs. IL-1β is a biomarker of immune exacerbations in leprosy (58). Interestingly, IL-1β production by MPs and DCs in response to *Candida albicans* signaling through Dectin-1 is regulated by Syk at two levels: transcriptional activation of pro-IL-1β and recruitment of the inflammasome via ROS and potassium efflux to produce mature IL-1β (38). Our data linking efficient CR3-mediated phagocytosis of *M. leprae*, or BCG producing PGL-I, and NFATc translocation to heighten production of IL-1β by MPs brings to the forefront a new regulatory mechanism. Although, we observed that Syk and CR3 regulated transcription of the *il-1b* gene, we did not rule out that NFATc translocation also regulated the inflammasome to produce mature IL-1β, which would be worth investigating. Therefore, our data highlight NFATc translocation to the nucleus, upon PGL-I targeting CR3 for phagocytosis, as a new pathway used to rewire the innate response against *M. leprae*.

It's also interesting to note that rBCG::PGL-I and rBCG::PGL-b are almost identical both in size and structure. They differ only by three sugars branched to the phenol nucleus of the PGL molecule (17). Strikingly, this small difference allowed sustained NFATc translocation, which highlights the key role of PGL-I in manipulating the innate immune system. PGL-I displays pleiotropic effects during leprosy infections: it down-modulates short-term TNF (17, 18) and iNOS (19) production by human MPs, it allows colonization of peripheral nerves via interaction with Schwann cells (14) and it initiates nerve damage by instructing NO producing MPs to patrol axons (16). In addition, PGLs from other pathogenic mycobacteria recruit growth-permissive monocytes (59) and allow escape from microbicidal MPs (60). Our data add a new weapon to this arsenal: by targeting CR3, PGL-I triggers the Syk/calcineurin/NFATc pathway that rewires the innate immune response in three major innate cells. We confirmed *in vivo* that rBCG::PGL-I allowed highly effective uptake by lung PMNs and MPs provided that CR3 was present and Syk active. Moreover, high levels of IL-10 were produced by lung cells infected with rBCG::PGL-I only when CR3 was present and this was abolished by NFATc inhibition. The lung must be considered as a portal of entry for the leprosy bacillus, since leprosy patients release contaminated droplets in nasal secretions that can be inhaled by contacts (5) and *M. leprae* interacts with epithelial lung cells that results in uptake by alveolar MPs and infection of the lung tissue in the mouse model (47). Interestingly, resident alveolar MPs upregulate CR3 expression under inflammatory conditions (48), which would also facilitate targeting by PGL-I upon *M. leprae* inhalation.

Thus, what could be the biological impact of NFATc nuclear translocation in the three major innate cells involved in modulation of the complex spectrum of the immune response to *M. leprae* (7)? During fungal infections PMNs rely on the NFATc pathway to efficiently kill pathogens and resolve inflammation through IL-10 production (41). In DCs, NFATc-induced IL-2 is essential to regulate DC half-life and avoid overwhelming inflammation (57) and CD103+ lung DCs, producing IL-2 via the NFATc pathway, regulate the local Th17 inflammatory response (42). In MPs, NFATc is retained in the cytosol by the kinase LRRK2 and *Lrrk2*^−/−^ mice develop severe colitis due to overwhelming inflammation upon NFATc translocation to the nucleus (61). Interestingly, leprosy patients carrying a *Lrrk2* missense variant are more prone to develop T1R with excessive inflammation (62) of which IL-1β is one biomarker (58). Our data that illustrate PGL-I/CR3 binding and NFATc translocation to generate high production of IL-1β by MPs could contribute to deciphering how excessive inflammation in severe leprosy takes place. NFATc also controls COX-2 expression leading to eicosanoid PGE2 production (45). We observed high levels of PGE2 production by DCs, MPs and PMNs upon infection with rBCG::PGL-I that was dependent of CR3 in agreement with efficient NFATc translocation to the nucleus. PGE2 can play both pro- or anti-inflammatory roles in leprosy depending on the clinical form (63). High levels of PGE2 secreted by Schwann cells and foamy MPs in LL may play an important role in Tregs induction. On the other hand, COX-2 is highly expressed in vessels and nerves of leprosy patients suffering from reversal reactions (64). IL-10 is abundant in lesions from LL patients and induces a specific phagocytic program by MPs (8). We showed that in mice PMNs were important cells for IL-10 production upon *M. leprae* surrogate, or native strain entry via CR3 and NFATc translocation to the nucleus. Whether PMNs are involved in rewiring the MP response in leprosy lesions would be interesting to determine. IL-10 is also highly detected during LL and is produced by Tregs and type 2 MPs (4). DCs producing IL-2 are known drivers of Tregs (65). Our data show that DCs highly upregulate IL-2 after *M. leprae* infection and NFATc translocation indicates that this pathway could also play a role in Treg induction in LL.

Leprosy ranks second in the order of human mycobacterial diseases and remains a threat in developing countries despite costly multidrug therapies programs. Management of reactions is crucial in preventing sensorimotor dysfunction in leprosy patients. In this respect, corticosteroids are recommended to relieve pain, inflammation and reversal of nerve damage (2) and thalidomide is highly effective to treat ENL (66). However, these treatments are hampered by side-effects including the disastrous teratogenic effects for thalidomide. Our work brings to the forefront NFATc as an important player in tuning the innate immune response to *M. leprae*. PGL-I that triggers this pathway upon CR3 binding, sustains IL-1β production by MPs that is coordinately regulated with PMN infiltration in ENL patients (67). Through IL-2 production by DCs sustaining Tregs (65) and PGE2 synthesis by MPs (63), NFATc translocation could also explain defective bacilli control in LL (68). Therefore, our work together with genetic profiling of leprosy patients (62) could help refine treatment of inflammatory reactions in leprosy by using NFATc blockers such as CsA (69) that have a long clinical track-record. This could improve quality of life of patients who develop intolerance to steroids and contribute to the global effort in preventing leprosy-associated disabilities in vulnerable populations.

## Data Availability Statement

The datasets generated for this study are available on request to the corresponding author.

## Ethics Statement

The animal study was reviewed and approved by Val de Loire Ethics Committee for Animal Experimentation (CEEA VdL) and registered by the French National Committee for Animal Experimentation.

## Author Contributions

ÉD-D designed and did most of the experiments, analyzed data, and prepared all manuscript figures. FC prepared all BCG strains and contributed to most experiments. AA did and analyzed the experiments to measure PGL molecules binding to human and mouse CR3. AR participated to cell infections studies and critically analyzed the data. ME realized flow cytometry analysis. WM contributed to critical reagents including construction of the fluorescent recombinant BCG strains. VM helped with cell-signaling experiments. JP synthesized the purified oligosaccharide domains for the PGL molecules. CA-D and CG supervised the work on PGL-I binding to CR3, critically analyzed the data and reviewed the manuscript. CD contributed to cell-signaling experiments, critically analyzed the data, and reviewed the manuscript. NW supervised all the aspects of the study, including execution of the experiments and wrote the manuscript.

### Conflict of Interest

The authors declare that the research was conducted in the absence of any commercial or financial relationships that could be construed as a potential conflict of interest.
