# Supplementary material for: CR3 Engaged by PGL-I Triggers Syk-Calcineurin-NFATc to Rewire the Innate Immune Response in Leprosy
Source: Front Immunol. 2019 Dec 17;10:2913. doi: 10.3389/fimmu.2019.02913 (PMC6928039; doi:10.3389/fimmu.2019.02913)
Supplement: Supplementary file 1 [file Data_Sheet_1.PDF]

Supplementary Figure S1

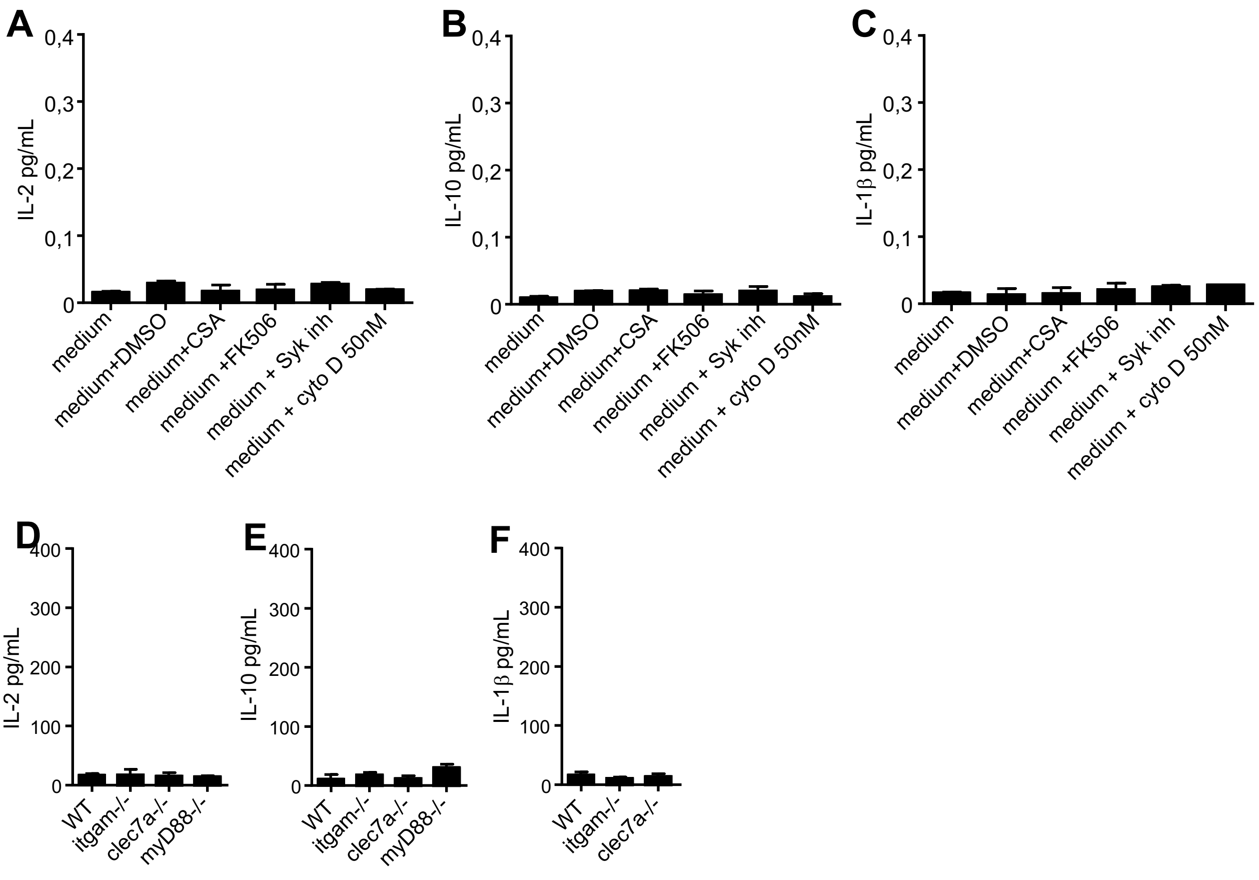

Background cytokine production by DCs, PMNs or MPs. Cells derived from bone-marrow of the different mouse lines were incubated with medium alone or with inhibitors or vehicle. Supernatants were harvested at the same time as in the different experiments performed with the mycobacterial strains. IL-2 produced by DCs, IL-10 by PMNs and IL-1b by MPs were measured by ELISA.
