# Supplementary material for: CR3 Engaged by PGL-I Triggers Syk-Calcineurin-NFATc to Rewire the Innate Immune Response in Leprosy
Source: Front Immunol. 2019 Dec 17;10:2913. doi: 10.3389/fimmu.2019.02913 (PMC6928039; doi:10.3389/fimmu.2019.02913)
Supplement: Supplementary file 2 [file Data_Sheet_2.PDF]

Supplementary Figure S2

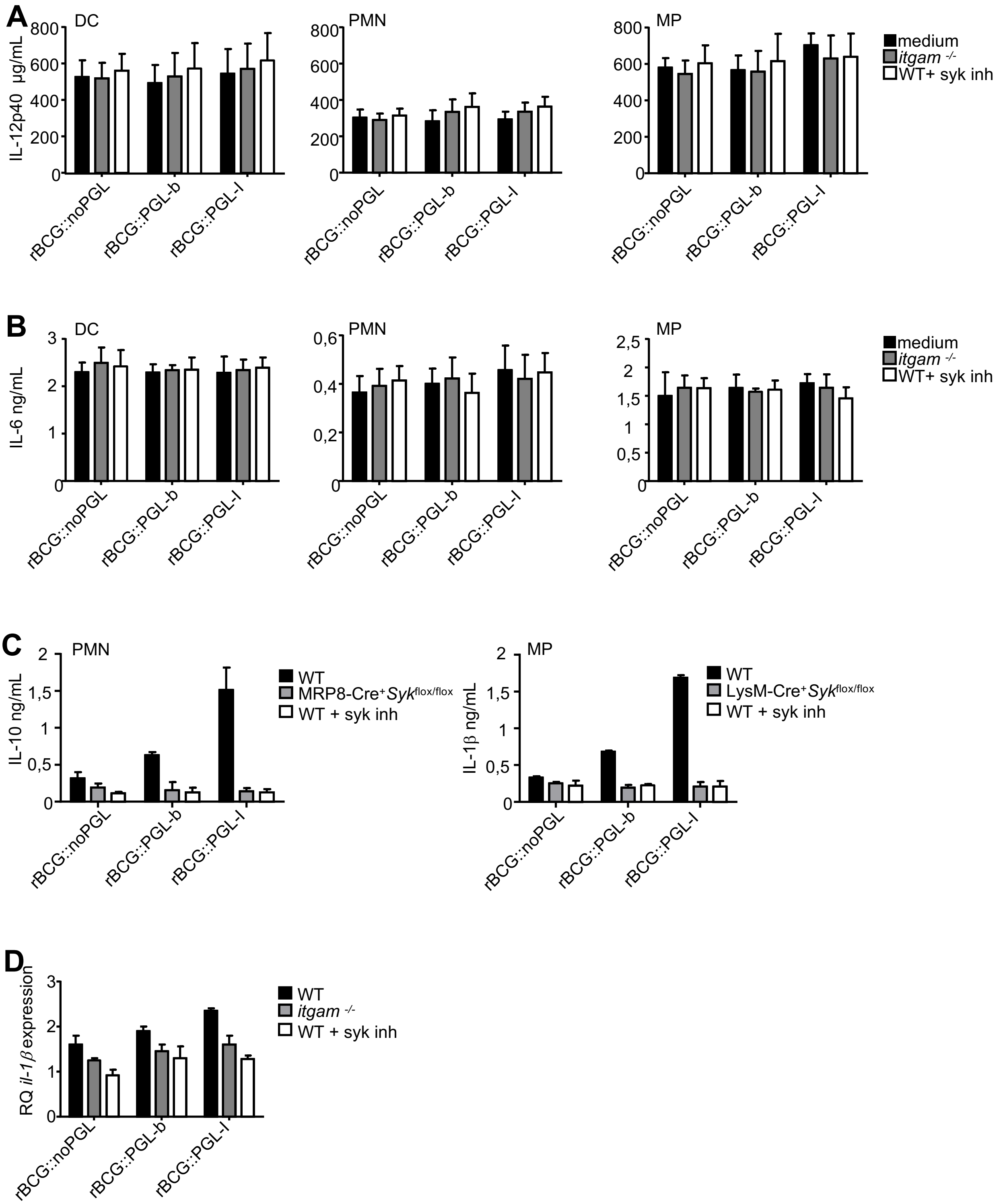

Targeting of CR3 by rBCG::PGL-I that engages Syk does not modulate NF-kB-dependent pro-inflammatory cytokines. DCs, PMNs and MPs derived from bone marrow of WT or *itgam*$^{-/-}$ mice were infected overnight with rBCG::noPGL, rBCG::PGL-b, or rBCG::PGL-I at MOI of 5. WT cells were treated with 1 μM of the Syk inhibitor GS 99-73, or DMSO as control. (**A**) IL-12p40 and (**B**) IL-6 produced in the supernatants were measured by ELISA. **(C)** Bone marrow cells were derived from MRP8-Cre+*Syk*$^{flox/flox}$ mice and MPs from LysM-Cre+*Syk*$^{flox/flox}$ mice to obtain PMNs or MPs genetically deficient for Syk, respectively. Cells and Syk proficient WT were infected with the three rBCG strains at MOI of 5. WT cells were also treated with 1 mM of the Syk inhibitor GS 99-73 for comparison. IL-10 secreted by in PMNs and IL-1β secreted by MPs after overnight incubation were measured by ELISA. (**D**) Evaluation by q-RT-PCR of *il-1b* gene expression by MPs derived from bone-marrow of WT or *itgam*$^{-/-}$ mice infected with the three rBCG strains and treated with DMSO or Syk inhibitor. Results are expressed as RQ ($2^{-\Delta\Delta Cq}$) with $\Delta\Delta Cq = \Delta Cq_{[infected\ cells]} - \Delta Cq_{[mock\text{-}infected\ cells]}$ after normalization to expression of three reference genes.
