# Supplementary material for: CR3 Engaged by PGL-I Triggers Syk-Calcineurin-NFATc to Rewire the Innate Immune Response in Leprosy
Source: Front Immunol. 2019 Dec 17;10:2913. doi: 10.3389/fimmu.2019.02913 (PMC6928039; doi:10.3389/fimmu.2019.02913)
Supplement: Supplementary file 3 [file Data_Sheet_3.PDF]

Supplementary Figure S3

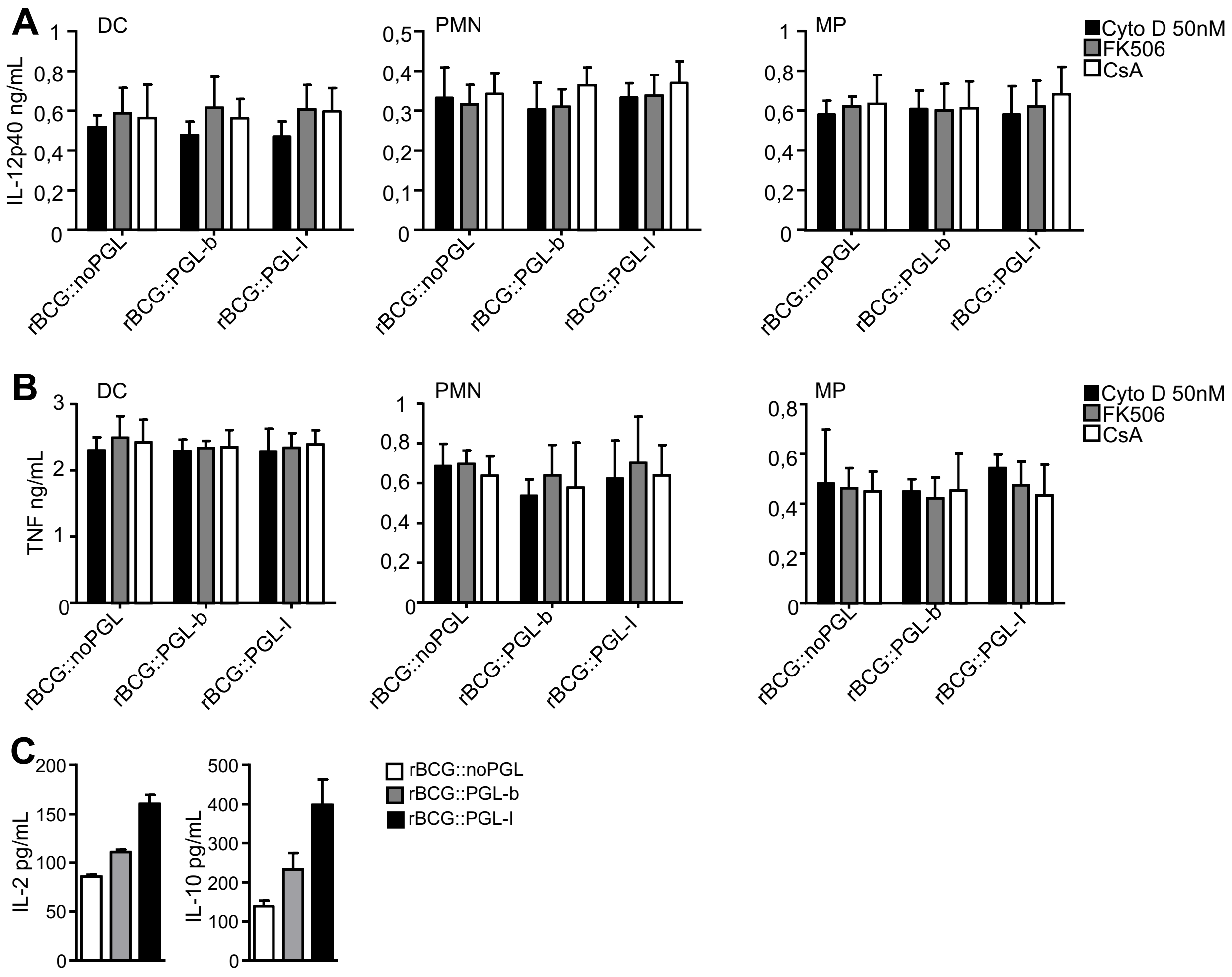

NFATc translocation does not regulate NF-κB-dependent pro-inflammatory cytokines
(**A, B**) WT DCs PMNs and MPs were treated for 1 h with the phagocytosis blocker CytoD (50 nM), or with the two NFATc blockers FK506 (500 pg/ml), or CsA (50 ng/ml) before infection with the three rBCG strains. IL-12p40 (**A**) or TNF (**B**) produced in the supernatant after overnight incubation were measured by ELISA. (**C**) Residual IL-2 and IL-10 produced by MYD88-deficient DCs and PMNs, respectively, after overnight infection with the three strains.
