# Supplementary material for: CR3 Engaged by PGL-I Triggers Syk-Calcineurin-NFATc to Rewire the Innate Immune Response in Leprosy
Source: Front Immunol. 2019 Dec 17;10:2913. doi: 10.3389/fimmu.2019.02913 (PMC6928039; doi:10.3389/fimmu.2019.02913)
Supplement: Supplementary file 4 [file Data_Sheet_4.PDF]

Supplementary Figure S4

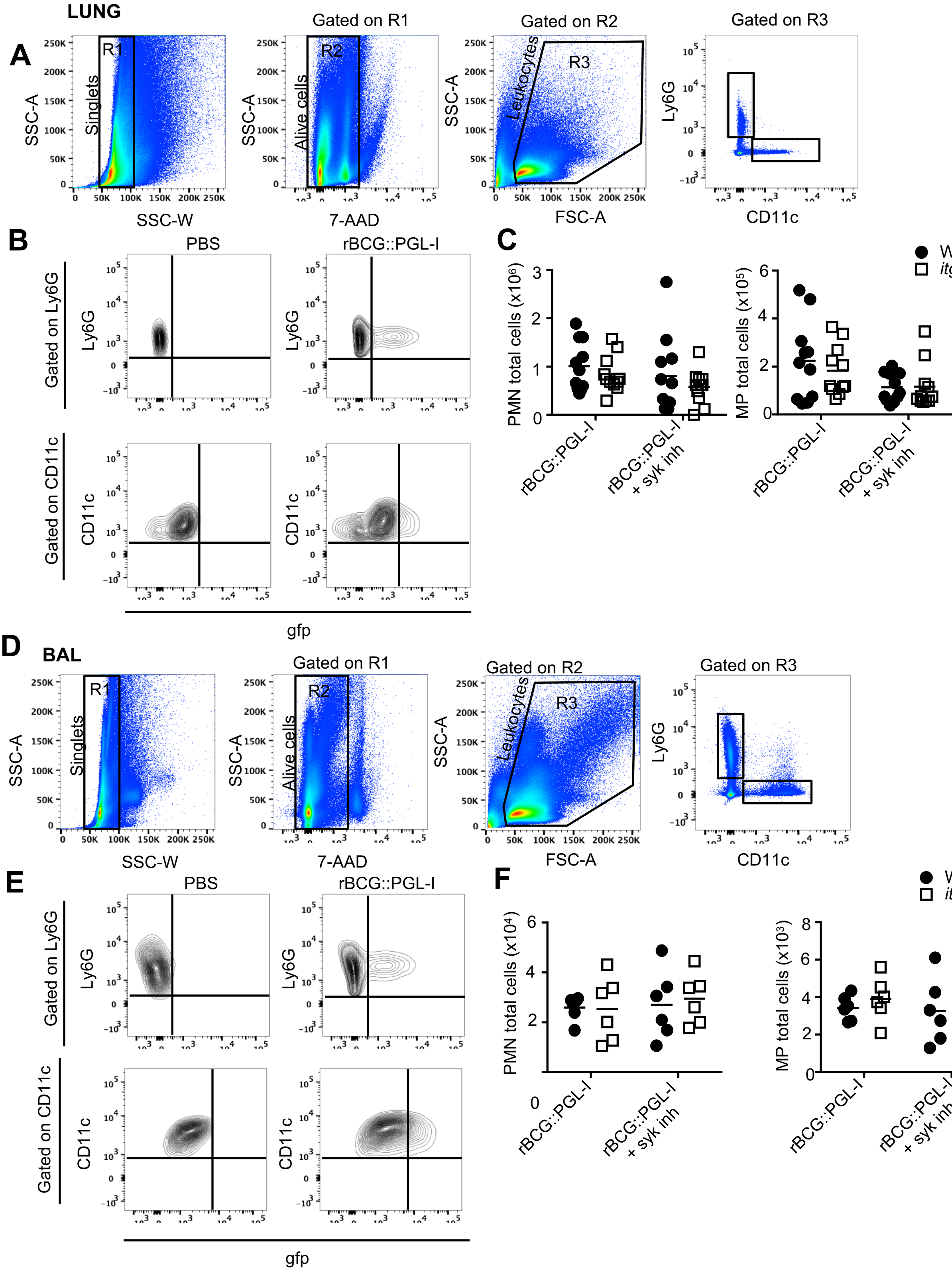

***In vivo* triggering of the Syk and NFATc pathway downstream of CR3**. WT and *itgam*$^{-/-}$ mice were nasally infected with 5x10^{6} CFUs of fluorescent rBCG::PGL-I or rBCG::noPGL. Mice received two nasal doses of 1 mM of the Syk inhibitor GS 99-73 1 h before and after infection. Cells from lung tissue (**A-C**) obtained after enzymatic treatment, or BAL (**D-F**) were harvested 24 h later and stained for 20 min with antibodies against the surface markers CD11b (clone M1/70), Ly6G (clone 1A8), Ly 6C (clone AL-21). Flow cytometry was realized with BD LSR Fortessa X20 flow cytometer equipped with 4 lasers: purple (405 nm), blue (488 nm), green/yellow (561 nm), red (640 nm) and 14 fluorescence detectors. (**A, D**) 10^{6} events were acquired for analysis with the following gating strategy: the R1 gate was set on singlets and 7AAD-positive dead cells were excluded to obtain gate R2. The R3 gate was set on myeloid cells in R2, based on their SSC and FSC profile and expression of the two surface markers CD11c and Ly-6G was analyzed to discriminate two cell populations: Ly-6G^{+} CD11c^{-} PMNs and Ly-6G^{-} CD11c^{+} MPs. (**B, E**) Representative dot plots of Ly-6G^{+} CD11c^{-} PMNs and Ly-6G^{-}CD11c^{+} MPs that acquired rBCG::PGL-I. Dot plots from mouse lung cells instilled with PBS are shown as controls. (**C, F**) Total numbers of Ly-6G^{+} CD11c^{-} PMNs and Ly-6G^{-} CD11c^{+} MPs recovered in the lung parenchyma from 11 individuals (**C**) or in BAL from 6 pools of 2 animals (**F**) are depicted.
