# Supplementary material for: CR3 Engaged by PGL-I Triggers Syk-Calcineurin-NFATc to Rewire the Innate Immune Response in Leprosy
Source: Front Immunol. 2019 Dec 17;10:2913. doi: 10.3389/fimmu.2019.02913 (PMC6928039; doi:10.3389/fimmu.2019.02913)
Supplement: Supplementary file 5 [file Data_Sheet_5.PDF]

Supplementary Table S1

A

	IL-2/DC						IL-10/PMN						IL-1β/MP					
	rBCG::no PGL		rBCG::PGL-b		rBCG::PGL-1		rBCG::no PGL		rBCG::PGL-b		rBCG::PGL-1		rBCG::no PGL		rBCG::PGL-b		rBCG::PGL-1	
clec7a -/-	77,4	±13,6	56,4	±10,4	56,2	±9,9	72,8	±12,5	57,8	±8,9	54,1	±9,7	52,5	±8,7	64,1	±2,7	57,3	±7,4
itgam -/-	80,5	±13,8	34,5*	±1,5	**27,2***	±3,2	74,2	±15,2	40,1*	±6,6	**15,5****	±3,7	67,7	±9,1	27,9*	±5,7	**18,3****	±7,6
syk inh	72,1	±9,2	31,1*	±1,3	**24,5***	±2,9	66,8	±13,7	38,3*	±1,7	**15,8****	±0,9	54,1	±7,3	22,3*	±4,6	**14,6****	±6,1

B

	IL-2/DC						IL-10/PMN						IL-1b/MP					
	rBCG::no PGL		rBCG:: PGL-b		rBCG ::PGL-1		rBCG::no PGL		rBCG:: PGL-b		rBCG ::PGL-1		rBCG::no PGL		rBCG:: PGL-b		rBCG ::PGL-1	
CytoD 1nM	59,22	±7,9	49,84	±3,9	51,70	±3,1	73,27	±11,2	44,03	±14,3	41,18	±6,1	55,43	*±14,0*	40,63	*±15,7*	47,65	*±4,9*
CytoD 50nM	33,93	±7,2	17,18**	±8,8	12,21*	±5,2	52,38	±21,1	54,46	±12,3	17,18**	±1,8	28,03	*±4,7*	23,08**	*±5,1*	7,95***	*±2,3*
FK506	54,01	±5,9	36,81**	±12,3	24,32**	±1,7	57,23	±18,2	43,44	±9,3	15,39**	±1,4	84,68	*±24,9*	26,77**	*±5,5*	13,31*	*±3,2*
CSA	67,80	±14,2	44,30*	±11,4	25,13**	±5,1	66,52	±19,5	55,49	±8,3	16,55**	±1,1	81,41	*±23,7*	34,61**	*±8,9*	11,56*	*±2,3*

C

	IL-2/DC						IL-10/PMN						IL-1β/MP					
	Syk inh		CsA		*itgam -/-*		Syk inh		CsA		*itgam -/-*		Syk inh		CsA		*itgam -/-*	
rBCG::PGL-1	19,23	*±4,9*	18,45	*±4,3*	18,51	*±5,2*	19,4	*±6,5*	16,4	*±2,5*	12,4	*±3,5*	20,6	*±3,7*	20,9	*±1,6*	19,3	*±0,9*
M. leprae	23,38	*±7,4*	23,77	*±3,5*	23,52	*±2,1*	26,6	*±6,4*	25,2	*±9,6*	14,6	*±6,5*	25,8	*±4,4*	24,3	*±5,4*	21,7	*±2,7*

Ratio of cytokines produced by treated versus control cells after infection with the different mycobacterial strains. DCs, PMNs or MPs were derived from bone marrow of the different mouse lines and infected with rBCG::PGL-1, rBCG::PGL-b or rBCG::noPGL at MOI of 5 or g-irradiated *M. leprae* (equivalent MOI of 10) under non-opsonizing conditions. As indicated, WT cells were also treated with inhibitors and control cells received DMSO. After overnight incubation, supernatants were harvested and indicated cytokines were measured by ELISA. Numbers in this table are ratio (%) of cytokine amount produced by drug-treated or receptor-deficient cells versus WT controls that were treated with DMSO when relevant. Data are presented as mean ± SEM (n= 4). In bold ratio differences that are statistically different as compared to controls after treatment of the data with paired non-parametric two-tailed K-Sample Fisher-Pitman Permutation test, with a Monte Carlo resampling approximation.
* $P < 0.05$; ** $P < 0.01$; *** $P < 0.001$.

A- DCs, PMNs or MPs were obtained from WT or *clec7a*$^{-/-}$ or *itgam*$^{-/-}$ mice. The Syk pathway was inhibited with 1mM GS 99-73.
B- DCs, PMNs or MPs obtained from WT were treated with 1 nM or 50 nM CytoD or the NFATc inhibitors FK506 (tacrolimus, 500 pg/ml) or CSA (50 ng/ml).
C- DCs, PMNs or MPs were obtained from WT or *itgam*$^{-/-}$ mice and stimulated either with rBCG::PGL-1 at MOI of 5 or g-irradiated *M. leprae* at equivalent MOI of 10.
